# Walking the good road of life: a longitudinal evaluation of American Indian youth suicide prevention training

**DOI:** 10.3389/fpubh.2025.1616464

**Published:** 2025-06-30

**Authors:** Clayton Small, Ernie Big Horn, Geri Small, Kellie Webb, Edwina Brown Bull, Maha Charani Small, Ruthie Cedar Face, Warren Pourier, Hawkeye Montileaux, Lance Christiansen, Brian Bradley, Wayne Trottier, Paola Trottier, Mike Geboe, Emily R. Beamon, Bethany Fatupaito, Brighten Crawford-Martin, Yolanda Ikazoboh, Kesiena Abeke, Sadie Posey, Kelley Milligan, Allyson Kelley

**Affiliations:** ^1^Native PRIDE, Fleming Island, FL, United States; ^2^Spotted Bull Resource Recovery Center, Poplar, MT, United States; ^3^Boys and Girls Club Northern Cheyenne Nation, Lame Deer, MT, United States; ^4^Doya Natsu Healing Center, Fort Washakie, WY, United States; ^5^Little Wound School, Kyle, SD, United States; ^6^Kayenta Unified School District, Kayenta, AZ, United States; ^7^New Town School District, New Town, ND, United States; ^8^Rocky Boy Tribal Health, Box Elder, MT, United States; ^9^Allyson Kelley & Associates PLLC, Sisters, OR, United States

**Keywords:** American Indian / Alaska Native, American Indian youth, suicide prevention, gatekeeper training, cultural intervention

## Abstract

**Background:**

American Indian/Alaska Native (AI/AN) populations have the highest suicide rate in the United States. Research on effective, culturally-centered, multi-level approaches to prevent suicide in AI/AN populations are limited.

**Methods:**

This multi-site longitudinal evaluation employed a retrospective pre-posttest design, utilizing a self-report survey administered daily following the training. Daily surveys included four areas related to suicide prevention, holistic wellness, generational knowledge, behavior change, and legacy impacts. The first objective of this study was to explore how the Good Road of Life training impacted participant knowledge regarding suicide and related risk factors while also exploring protective behaviors and impacts from a culturally-centered, strengths-based lens. A second objective was to present a conceptual model grounded in socioecological and trans-ecological theories of GRL for collective healing targeting the individual, family, community, Tribe, and nation to prevent suicide.

**Results:**

Between 2019 and 2024, 27 GRL trainings were conducted at 8 Tribal sites in Montana, South Dakota, North Dakota, and Wyoming. Data accumulated across these 27 trainings and 1,810 students represented diverse tribes, locations, and ages. Students were asked to rate different domains of Holistic Wellness: mental (*M* = 3.25, *SD* = 1.03), physical (*M* = 3.33, *SD* = 1.03), spiritual (*M* = 3.35, *SD* = 1.05), and emotional (*M* = 3.17, *SD* = 1.12). Students rated 17 generational knowledge domains before and after GRL training; all comparisons were significant at *p* < 0.001; all differences had moderate or large effect sizes. Middle school students reported larger increases in knowledge from pre to post than high school students.

**Discussion:**

Findings from this evaluation underscore the importance of early intervention, culturally-centered approaches, and community-led suicide prevention in AI/AN populations.

## Introduction

American Indian and Alaska Native (AI/AN) populations are rich in culture, traditions, kinship, and community. With 574 unique federally recognized Tribal sovereign nations in the United States, each has its distinct history, language, culture, and story. Prior to colonization, AI/AN children learned about traditional ways of life from their immediate and extended family members. Elders and families would talk with young people in their Native language and use words that helped their child feel loved and accepted. In these teachings, children also learned to respect others. Extended family members, such as aunts and uncles, were often responsible for teaching children about safety, self-care, and healthy relationships. Grandparents taught young people about life, how to speak in a good way, and how to conduct themselves; they were also responsible for passing down traditional knowledge and family kinship systems that supported holistic wellbeing (1).

Colonization, by way of Reservation systems, residential boarding schools, and foster care systems, disrupted traditional roles and teachings within AI/AN family kinship systems and resulted in cultural genocide. Colonial impacts and intergenerational traumas create conditions where AI/AN communities experience higher rates of suicide and lower life expectancy (2). Created in 1851, the United States government’s reservation system confined Native people to reservations, without access to traditional foods and hunting; as a result, many died and experienced severe malnutrition. Reservations face similar conditions today, characterized by limited economic opportunities, persistent poverty, and vast structural inequities that threaten the vitality and longevity of AI/AN people (40). The Native American residential boarding school movement, established in the late 1800s by Christian missionaries, aimed to “Kill the Indian and Save the Man.” Native children were forcibly removed from their homes and placed in residential boarding schools, as young as 4 years old. They were forbidden to speak their traditional language and could not wear traditional clothing; their long hair was cut short, and they were not allowed to use their Indian names. The physical, sexual, psychological, and emotional abuses that occurred in boarding schools linger in the hearts and minds of many Native families today, creating soul wounds and intergenerational traumas (3). Native foster care programs starting in the late 1800s removed Native children from their families and placed them in the care of non-Natives. Forced removal and foster care continue to negatively impact AI/AN family kinship systems, with Native children being 11 times more likely to be placed in foster care than a white child (41).

This study contributes new knowledge about the effectiveness of a culturally-centered curriculum in strengthening protective factors associated with suicide in AI/AN populations.

### Background

Western, medicalized approaches to address colonial impacts and prevent suicides in AI/AN populations are not effective. AI/AN people have the highest rates of suicide in the nation, more than any other racial or ethnic group (4). Suicide is the second leading cause of death among AI/AN adolescents (5), with a crude suicide rate of 18.95 per 100,000—this is 170% more than that of non-Hispanic white adolescents (6). AI/AN youth data from the 2021 US Centers for Disease Control and Prevention Youth Risk Behavior Survey (YRBS) indicated that 27% of AI/AN youth had considered attempting suicide, 22% planned suicide, 16% attempted suicide, and 1% were injured due to suicide attempts (6). YRBS data also indicated there were 65 AI/AN adolescent suicides in 2021; of these, 40 were male, and 25 were female. Sexual minority status (SMY) was not reported in any AI/AN suicides during this period; however, SMY AI/AN youth are more likely to have suicidal thoughts and previous attempts than non-SMY AI/AN youth (7). Research studies and national surveys like the YRBS may pathologize AI/AN people and create a suicide narrative about who they are and what they experience. Suicide deficit-based narratives are problematic because they can create self-fulfilling prophecies and messages- that AI/AN people are at risk and complete suicide at higher rates than others and that they “need to be saved” (39). This colonial “Save the Indian” mentality is evident in deficit-focused narratives and perpetuated with efforts from national agencies like the Substance Abuse and Mental Health Services Administration (SAMHSA) and the Native Connections Behavioral Health grant that funds Tribes and communities with the highest rates of suicide. Approaches that focus narrowly on preventing suicide without addressing multiple levels of risk and protective factors (past, present, and future) are not effective with AI/AN populations.

Existing literature outlines known suicide risks and protective factors in AI/AN populations. Suicide is often narrowly viewed as a psychological problem (Walkup et al., 2015) with individual risk factors that include depression, hopelessness, poor mental health, peer rejection, trauma, substance use, biological sex, interpersonal conflict, and alcohol use (8). Among the factors that influence suicide risk, *gender* is a major factor. AI/AN females are more likely to experience suicidal ideation, but AI/AN adolescent males are more likely to complete suicide attempts (9). Experiences of *racism and discrimination* have also been implicated as risk factors for suicidal behaviors in AI/AN adolescents (42). *Forgiveness* and one’s ability to forgive others relates to lower levels of suicidal behavior—when individuals can forgive themselves and others, this results in reduced stress and a greater sense of self-control and ability to reestablish social relationships (10). Feeling *hopeful and positive* about the future, relying on sources of strength, and having access to healthy peers and adults are protective factors that may mitigate suicide risk (11). These individual risk factors often become the focus of suicide prevention and intervention strategies. This is problematic because in many AI/AN communities, suicide is not viewed as an *individual* problem, but a *collective* issue that is influenced by social, cultural, and historical contexts (12). Within most AI/AN communities, there exists collective shame, guilt, and burden; however, there is also collective healing, resilience, and wellbeing. One individual loss is felt collectively by the entire community. Similarly, gains in knowledge, healing, and wellbeing, are collectively shared and felt throughout a family, community, and future generations. An emerging body of literature focuses on the social and structural factors related to suicide risk, which include the lingering impacts of colonization, social destruction, international trauma, and persistent oppression (13). At the community level, social determinants of health can increase the risk of suicide- high rates of unemployment, adverse childhood experiences, feeling unsafe at school and home, collective grief, limited social capital, poor housing, limited quality education, and limited access to healthcare providers (12). Geographical location increases risk, as AI/AN youth living on a reservation are up to six times more likely to die from suicide than non-reservation youth (13).

Cultural factors have not been widely studied regarding suicide and often vary based on AI/AN history, traditions, and protocols. However, some reports show that cultural commitment, awareness of Tribal traditional beliefs about suicide, spirituality, historical loss, traditional practices, and cultural health values contribute to suicide risk or protection (8). In other studies, community support, connection to language and land, and psychological resilience have been viewed as unique social and cultural assets, buffering the risk of suicide (13). Being connected to one’s family and the ability to discuss problems with family and friends may also attenuate the risk of suicidal behaviors in AI/AN youth (14).

A key issue in addressing suicide with AI/AN populations is how it is defined and conceptualized. Within Western scientific literature and medicine, suicide is defined as intentionally ending one’s own life (15). AI/AN communities can have different concepts of suicide; in many tribes, suicide is not a word in the cultural vocabulary (16). The AI/AN definitions of suicide can also vary; some view it as a spirit that comes to help people and communities in pain (17). The ways that AI/AN communities define and conceptualize suicide go beyond one diagnosis, one risk factor, or one intervening variable and have direct implications for how approaches are designed to prevent, intervene, and strengthen community conditions to address suicide. Thus, in AI/AN communities, interventions designed to address suicide should incorporate cultural connectedness, identity, kinship systems, spirituality, and ceremonies, while addressing the political, social, and historical factors that place AI/AN communities at a greater risk (8). Such approaches and responses need to originate within AI/AN communities rather than being led by external agencies and non-AI/AN groups that are unfamiliar with the culture, language, kinship systems, history, context, or needs (11). The use of AI/AN community-led approaches is consistent with the 2024 National Strategy for Suicide Prevention, Strategic Direction 1, which calls for integrating and sustaining community-based prevention (18) and previous research, policy, and program recommendations (19, 20).

One commonly used approach to prevent suicide in AI/AN communities is gatekeeper training. Gatekeeper trainings teach individuals how to recognize suicide risk factors and get the people in need of help and the resources they need. However, research on the effectiveness of AI/AN suicide prevention gatekeeper trainings to prevent suicide is limited. American Indian Life Skills (AILS) is a suicide prevention and life skills training program aimed at reducing behaviors and cognitive factors associated with suicidal behavior (21). Previous studies show AILS is effective in reducing hopelessness, increasing confidence, and intervening with suicidal behaviors (21). Online and text-messaging-based gatekeeper training programs (such as Mind4Health) are increasingly utilized to develop gatekeeper skills in mental health conversations and enhance knowledge of referrals to community resources (22). Programs like Applied Suicide Intervention Skills Training (ASIST), Question, Persuade, Respond (QPR), and Suicide Alertness for Everyone (SafeTALK) are evidence-based suicide prevention trainings (23) designed to prevent suicide. The effectiveness of ASIST and SafeTALK to address suicide in AI/AN populations is also not well documented. Critics feel that colonized gatekeeper trainings conflict with AI/AN epistemologies and often fail to incorporate Tribal context, community, and culture into their overall approach (11, 24, 25).

### Purpose

AI/AN scholars, policymakers, and elders call for holistic and culturally-centered practices in all suicide prevention and gatekeeper trainings while recognizing that collective grief, trauma, unresolved loss, and persistent oppression contribute to conditions that increase the risk of suicide (20). The Good Road of Life (GRL) curriculum responds to this call, offering a culture- and resilience-based, community-led, suicide prevention curriculum that leverages the strengths that Native people possess. Previous publications of the GRL curriculum document its positive impacts among American Indian youth, American Indian fathers, mental health workers, and other members of the American Indian community (11, 26). However, no longitudinal study or extensive multi-site evaluation of the GRL has been conducted, resulting in a gap in the literature regarding its effectiveness in strengthening protective factors associated with suicide.

This study aimed to investigate the effects of GRL training on American Indian youth across multiple sites within the country. From 2019 to 2024, data from this study represents 8 Tribal communities and consists of a broad representation of gender, many ages, and varying locations. The first objective of this evaluation was to explore how GRL impacted participant knowledge regarding suicide and related risk factors while also exploring protective behaviors and impacts from a culturally-centered, strengths-based lens. A second objective was to present a conceptual model grounded in socioecological and trans-ecological theories (12, 26) of GRL for collective healing targeting the individual, family, community, Tribe, and nation to prevent suicide.

### About the Native PRIDE GRL curriculum

The GRL curriculum is supported by a 225-page training manual that each participant receives and uses throughout the entirety of their training. Training is typically offered over 3 days, spanning approximately 18 h. Chapter topics address suicide and include the following: social norms, curriculum overview, clan formation, colonization and racism, multigenerational trauma and breaking unhealthy cycles, sobriety, hostility and anger management, domestic violence, healing, forgiveness, grief, suicide prevention, sexual orientation, conflict resolution and healthy communication skills, and goal setting. Culture is a significant factor in GRL training, often incorporating elements such as local drum groups, storytelling, equine wellness, naming ceremonies, visits to sacred sites, traditional dancers, elders serving as clan leaders, and a sweat lodge. These activities vary and are driven by communities during the planning stage. Importantly, GRL is tailored to the community, culture, and needs. For example, when GRL training is offered in a school setting (following a youth suicide), the topics also focus on self-care, recognizing the signs of suicide, and developing peer helper skills. Students also learn about suicide prevention resources in their communities. Expected outcomes from attending the GRL include developing skills to manage stress, increasing cultural resilience, enhancing communication and conflict resolution, reducing suicide risk factors, improving peer counseling skills, developing leadership skills, and promoting empowered and healthy decision-making.

The non-profit organization of “Native PRIDE” aims to develop and implement culture, strengths, and spiritual-based programs for Native people that inspire leadership, healing, and wellness from colonization and multigenerational trauma. Their philosophy is to be “A Good Relative” in the facilitation of wellness and healing for Native youth, families, communities, and organizations. Unlike other gatekeeper trainings, GRL is only offered when an American Indian community requests it via the Native PRIDE website,^1^ email communications, or in-person interactions. Native PRIDE collaborates closely with reservation-based and non-reservation-based organizations and schools to recruit participants and implement the training. Planning GRL training begins with determining the focus population, the reasons for requesting a GRL, and the time and location of the GRL. Dr. Clayton Small collaborates with organizations and schools, including principals, teachers, cultural leaders, mental health professionals, and others, to develop GRL training plans. Training enrollment is open to American Indian youth between the ages of 6 and 18 who are students at a participating school or organization. Typically, the GRL includes a 1-day Training of Trainers, which increases community capacity to respond to suicides once training has been completed. Trainers are often mentors, teachers, and leaders in their communities who have been identified as individuals with the skills, knowledge, and abilities to implement GRL training successfully over time. It is then followed by a 3-day GRL training for students. Recruitment of GRL participants varies based on the location of the GRL training. For example, in schools, recruitment is not necessary because the GRL is offered during regular school hours. Students have an opportunity to opt out of the training if they do not wish to attend. Recruitment follows established protocols and guidelines in health-related programs or youth-serving organizations. This may include posting flyers, engaging with partners and caregivers, and conducting in-person communications regarding the GRL training. When GRL is offered immediately following a suicide completion or cluster recruitment, it follows similar tribally-designated protocols, being sensitive and responsive to the community needs and conditions. GRL is consistently implemented by its developer, Dr. Clayton Small, resulting in a high level of fidelity to the GRL curriculum and consistent delivery within each community.

## Methods

### Setting

This evaluation was conducted by Native PRIDE, a 501(c)3 in partnership with Allyson Kelley & Associates, PLLC (AKA). Between 2019 and 2024, 27 GRL trainings were conducted at 8 Tribal sites, including six schools, one youth-serving organization, and one community-based healing center. In order to document the effectiveness of the GRL training, data accumulated across these 27 trainings were aggregated to create a larger, analytical dataset. Data included both quantitative survey responses and qualitative data from open-text questions (see Supplementary file). Over the course of 5 years, through developments in training and evaluation techniques, students were not asked the same demographic or construct-specific items. As a result, while the entire dataset includes 1,810 students, the sample sizes for each demographic characteristic and each knowledge construct will differ.

### Procedures

This multi-site longitudinal evaluation employed a retrospective pre-posttest design (27), utilizing a self-report survey administered daily following the training. Students who completed evaluations were entered into a raffle for gift cards, t-shirts, and other desirable items. Response rates varied by location; some reported response rates of 50%, while others reported 100%. Surveys were not required to be completed to participate in the GRL training. Written child assent and parental permission were obtained for participants under 18. Evaluation of the GRL trainings followed Tribal research and evaluation protocols in each community as a sovereign nation. Tribal councils, school administrators, and teachers reviewed and approved all evaluations. When GRL trainings were offered to students in grades K-6, questions were modified, adapted, or in some cases eliminated (see Supplementary file).

### Participants

Participants were recruited using a convenience sample of students who attended each GRL training session over 5 years and 27 training sessions. Students were given the option to participate (or not) in the evaluation. Recruitment followed GRL methods mentioned previously, including working with local schools and youth serving organizations to schedule trainings, post flyers, and engage with parents and caregivers of student participants. The dataset for this study was created by aggregating survey information from all 27 GRL trainings across 5 years of the evaluation.

Among the total number of students surveyed across 5 years, not all participants were asked the same items every time. Throughout the course of evaluating the GRL training, items were included/excluded from surveys due to a number of factors: changes in the sampling of students (e.g., boys groups only, elementary v middle school v high school students), different sites of training across the United States, changes in the curriculum being taught [training could be requested and adjusted following an important event (i.e., suicide) in the community], or adjustments to the evaluation itself. Because of the evolving nature of the evaluation, there were variables (demographic and outcome variables) that were completed by some students and not others.

The demographic characteristics for GRL training varied by training location and community (see Table 1). Within the entire dataset (*N* = 1,810), students ranged in age from 6 to 18 years, most students were female, there was equal representation from middle and high schools, and of students explicitly asked, 81% reported being American Indian. Within the smaller subset of data that was used for all analyses (*N* = 1,550), students ranged in age from 10 to 18, most students were female, most were in middle school, and most reported being AI/AN.

**Table 1:** Demographic characteristics of GRL youth participants (*n* = 1,810).

Demographic	n (%)
Age (*N* = 1,078)	Range = 6–18 Median = 13
Gender (*N* = 1,136)	
Female	578 (51)
Male	538 (47)
Non-binary / Third Gender	18 (2)
Prefer Not to Say	2 (<1)
Grade (*N* = 1,716)	
Elementary School	172 (10)
Middle School	787 (46)
High School	757 (44)
American Indian / Alaska Native (*N* = 374)	
Yes	303 (81)
No	71 (19)
Location (*N* = 1,430)	
Dunseith, ND	186 (13)
New Town, ND	483 (34)
Oberon, ND	83 (6)
Kyle, SD	205 (14)
Ethete, WY	96 (7)
Hardin, MT	69 (5)
Lame Deer, MT	202 (14)
Poplar, MT	106 (7)

Sample sizes for each demographic variable will change—not all demographic characteristics were asked of all participants; the table reflects the entire data set.

### Instrumentation

The initial GRL survey was developed by Native PRIDE and AKA in partnership with Tribal communities; it was reviewed, edited, and updated over a 10-year period. Early reports, publications, and evaluations of the GRL evaluations demonstrate the progress and evolution of GRL data collection over time (11). The GRL survey instrument utilizes single-item outcomes, and all questions were validated for cultural context and intent by Tribal partners, authors, and previous participants. Surveys asked for limited demographic information to protect the anonymity of students in small communities. Daily surveys included four areas related to suicide prevention, holistic wellness, generational knowledge, behavior change, and legacy impacts. Other questions asked students to rate their satisfaction with the training and facilitators (see Supplementary file) or, in some cases, site-specific questions related to leadership, emotional intelligence, strengths, culture, and other areas. These questions and responses are not included in this publication.

Holistic Wellness. Using the medicine wheel (28) as a guide, questions evaluated student wellness ratings using mental, physical, spiritual, and emotional domains. Data for holistic wellness were collected from four items that asked, “Rate your mental, physical, spiritual, or emotional wellness,” using a 5-point Likert scale (*1 = poor, 5 = excellent*).

Generational Knowledge & Understanding. Questions evaluated student knowledge and understanding of 17 protective constructs before and after the training. These questions were developed based on the GRL curriculum goals and objectives. Data for knowledge scores were derived from mean composite scores in response to knowledge questions, which used a 10-point scale (*1 = no understanding, 10 = complete understanding*). Daily knowledge questions and surveys corresponded with the GRL daily agendas and content delivered. In most cases, daily agendas were similar across sites, but there was some variation, which is notable in the sample sizes reported (see Table 2 for the list of knowledge areas).

**Table 2:** Knowledge and understanding construct comparisons.

Variable	n	Before	After	T Value	Cohen’s D
Grief & Loss	620	5.98	7.90	−18.48	−0.742
Suicide Prevention	589	6.12	8.10	−19.48	−0.802
Spirituality	403	5.82	7.90	−18.92	−0.942
Team Building	358	5.97	8.32	−18.44	−0.974
Forgiveness & Letting Go	353	5.29	8.04	−19.16	−1.02
Establishing & Maintaining Sobriety	305	5.93	8.03	−15.11	−0.865
Conflict Resolution	297	5.68	7.79	−15.47	−0.898
Colonization, Racism, and the Role of Native People	268	5.44	7.76	−15.69	−0.958
Sources of Strength	263	6.08	7.76	−11.63	−0.717
Healthy Relationships	239	5.89	7.99	−13.87	−0.897
Multigenerational Trauma and Breaking Unhealthy Cycles	171	6.01	7.61	−8.71	−0.666
Consequences of Substance Abuse	162	6.52	8.06	−8.92	−0.701
Healthy Communication Skills	153	6.40	7.93	−9.55	−0.772
Hostility & Anger Management	149	6.43	7.93	−8.41	−0.689
Historical Trauma	142	5.40	7.61	−12.28	−1.03
Coping with Trauma	88	6.24	8.02	−5.96	−0.635
Healing Shame & Addressing Forgiveness	76	6.38	7.82	−5.78	−0.663

All differences are significant at *p* < 0.001.

Behavior Change. Using open-text response questions, students were asked, “Will you change anything as a result of this training?” where response options were yes, maybe, or no. A follow-up question was asked to students who selected yes: “What will you change as a result of this GRL training?” Responses were open text. These questions were developed based on the theory of planned behavior (29) and previous GRL evaluations that utilized similar question formats to document intended behavior changes.

Legacy Impacts. Using early GRL evaluations (11) to formulate a list of possible impacts, students were asked to select all the ways they were impacted by the GRL training. Response options included being more hopeful, breaking unhealthy cycles, goal setting, understanding the impacts of colonization, helping someone who is suicidal, feeling connected to spirit, addressing conflicts, knowing forgiveness, dealing with grief, and understanding the impacts of historical trauma and racism.

### Data analysis

Data were analyzed using SPSS Version 29.0 and examined the effect of GRL trainings on 17 protective constructs that are taught within the GRL curriculum. From the aggregated sample (*N* = 1,810), participants were selected for analyses if they completed key demographic variables (grade, gender, or age), and had responses to most of the outcome variables. As a result, the sample size within analyses varies as a function of how often the variables were asked, and when they were added to the repertoire of the evaluation. Analyses were conducted in their respective topic for participants with the most complete data. Consideration of non-response bias was considered; however, most of the students in attendance at the training did complete the survey. Early versus late survey bias was addressed by having all students complete the evaluation on the last day of training.

Paired-samples t-tests were conducted to examine the differences in student levels of understanding from pre to post-GRL trainings; power analyses indicated that we would need at least 25 pairs to achieve 80% power for this study, with an anticipated effect size of 0.60. Independent samples t-tests analyzed the differences in demographic characteristics across all dependent variables (power analyses indicated a sample size of 90 to achieve adequate power for this study). Factorial models were used to consider multiple independent variables on pre-post difference scores; power analyses suggested groups of at least 30 to achieve 80% power with effect sizes of 0.60. Qualitative data from behavior change open-text responses were analyzed using thematic analysis methods (30). Consistent with a thematic approach that is unbounded, this involved the examination of recurring themes and patterns of meaning using an inductive approach. First, the evaluation team became familiar with the data. The qualitative analysis process involved hand-coding techniques and team meetings to discuss and code textual data. One evaluation team member generated a list of initial codes from open-text questions using hand coding techniques and an Excel spreadsheet. Next, the team member searched for themes, reviewed them, defined and named themes. This was followed by the interpretation of themes within the qualitative data presented. Qualitative themes were validated by two members of the evaluation team and also confirmed by GRL sites and co-authors of this paper. No discrepancies were found in the identified themes.

## Results

*“Today was good but sad. I had to talk about my mom’s death.”*—GRL Student Participant.

**Figure 1:**
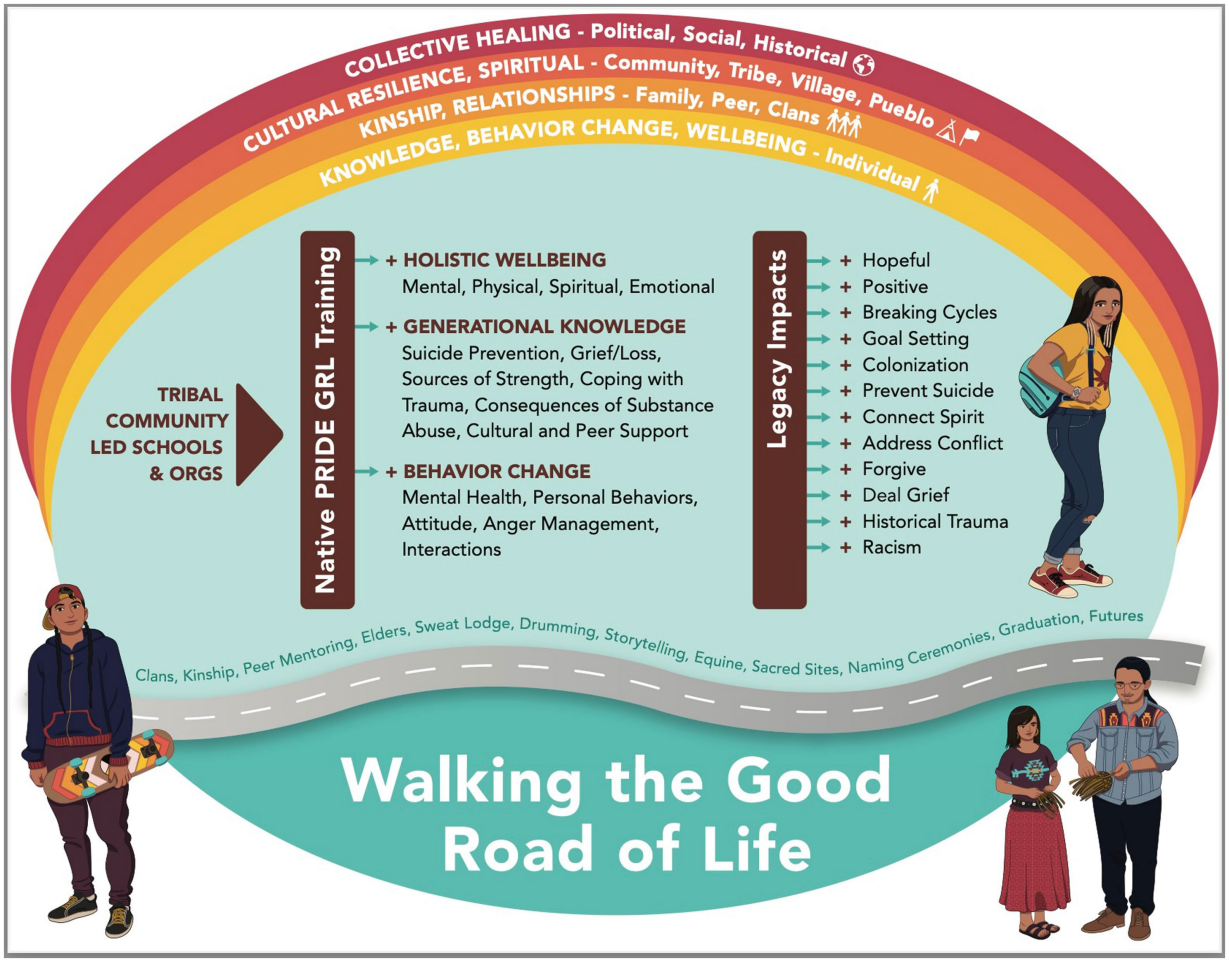
Conceptual model of GRL for collective healing.

GRL addresses multiple levels of wellbeing that promote resilience and culture to walk the Good Road of Life. Tribal communities, schools, and organizations initiate GRL. Components of the GRL training lead to holistic wellbeing, increases in generational knowledge, and positive behavior change. The legacy impacts of the GRL training influence individuals, families, communities, and histories (see Figure 1).

### Holistic wellness

Students were asked to rate different domains of Holistic Wellness: mental (*M* = 3.25, *SD* = 1.03), physical (*M* = 3.33, *SD* = 1.03), spiritual (*M* = 3.35, *SD* = 1.05), and emotional (*M* = 3.17, *SD* = 1.12). Overall, students rated their wellness positively, with less than 6% reporting any of these domains as “poor.” All domains positively correlated with each other at levels of at least 0.50 and above.

When examining gender differences among holistic wellness domains, there were no differences for mental wellness or spiritual wellness (see Table 3). However, male students rated their emotional and physical wellness higher than their female counterparts. Holistic wellness domains based on American Indian status were not significantly different.

**Table 3:** Domains of holistic wellness by gender.

Domain of holistic wellness	Female M (SD)	Male M (SD)	T value	*p* value	Cohen’s D
Mental Wellness (*n* = 397)	3.22 (0.92)	3.37 (1.06)	1.55	NS	N/A
Spiritual Wellness (*n* = 395)	3.38 (0.98)	3.37 (1.06)	−0.07	NS	N/A
Emotional Wellness (*n* = 396)	3.13 (1.04)	3.48 (1.07)	3.33	< 0.001***	0.34
Physical Wellness (*n* = 397)	3.26 (0.96)	3.63 (0.96)	3.70	< 0.001***	0.37

***p < 0.001; **p < 0.01. there is no.

### Generational knowledge and understanding

Students were asked to rate their level of knowledge and understanding for each of the following protective constructs before and after GRL training (see Table 2). All comparisons were significant at *p* < 0.001; all differences had moderate or large effect sizes.

A difference score was calculated between the pre- and post-Knowledge & Understanding scores on each of the 17 domains. These scores were then correlated with the holistic wellness domains. All 17 constructs were positively correlated with each of the following wellness domains. This suggested that as the student rated their holistic wellness scores higher, they experienced *greater increases* from pre to post scores on their respective construct. Higher Mental Wellness scores were correlated to greater increases in scores of Coping with Trauma (*r* = 0.42, *p* = 0.03), Establishing & Maintaining Sobriety (*r* = 0.25, *p* = 0.02), and Forgiveness & Letting Go (*r* = 0.18, *p* = 0.02). Higher self-reported Physical Wellness scores correlated with larger difference scores from pre to post on Conflict Resolution (*r* = 0.26, *p* = 0.03) and Sources of Strength (*r* = 0.22, *p* = 0.03). Higher Spiritual Wellness scores correlated with larger difference scores from pre to post on Colonization, Racism, and the Role of Native People (*r* = 0.19, *p* = 0.04). Higher Emotional Wellness scores were correlated with larger differences on the construct of Healing Shame (*r* = 0.55, *p* = 0.01).

### Middle and high school comparisons

Among the data that provided the age of the student, there were no relationships directly with age and holistic wellness domains. However, age negatively correlated with the difference scores of Coping with Trauma (*r* = −0.351, *p* < 0.001), Establishing & Maintaining Sobriety (*r* = −0.209, *p* = 0.001), and Conflict Resolution (*r* = −0.139, *p* = 0.04). As the age of the student decreased, their scores from pre to post got larger; younger students experienced greater increases from their pre to post scores with these specific constructs.

Across all datasets, the student’s age was not always available; however, we were able to determine the level of schooling for students in lieu of their exact age. Analyses were conducted comparing middle—to high school-aged GRL students. There were no significant differences between middle and high school students on holistic wellness domains.

Concurrently considering the level of schooling *and* gender of the students, there were only significant effects for the holistic wellness domains of Physical and Emotional Wellness. We conducted a 2 (level of schooling) x 2 (gender) ANOVA and found a significant main effect for gender with Emotional Wellness, *F* (1, 392) = 12.20, *p* < 0.001, *partial eta^2^* = 0.03 (means for this main effect are captured above in Table 3). There was no main effect for level of schooling or interaction for Emotional Wellness.

There were two main effects for both independent variables (level of schooling and gender) with regard to Physical Wellness. Males rated their physical wellness greater than females, *F* (1, 393) = 16.48, *p* < 0.001, *partial eta^2^* = 0.04 (see Table 3 with descriptive statistics for gender); high school students (*M* = 3.50, *SD* = 0.98) also rated their physical wellness greater than middle school students (*M* = 3.37, *SD* = 0.99), *F* (1, 393) = 3.97, *p* < 0.05, *partial eta^2^* = 0.01; there was no interaction for level of schooling by gender.

The level of schooling and gender on the aforementioned construct difference scores using a 2 (level of schooling) x 2 (gender) ANOVA also revealed effects on four of the Knowledge and Understanding difference scores. With regard to the construct of Forgiveness and Letting Go, there was a main effect for gender, such that female students (*M* = 3.11, *SD* = 2.37), regardless of grade level, experienced higher increases from pre to post on this construct than male students (*M* = 2.25, *SD* = 2.68); there were no other main effects or interactions.

There were main effects for level of schooling on difference scores of Establishing & Maintaining Sobriety, Conflict Resolution, and Healthy Relationships (see Table 4); there were no other main effects or interactions for these constructs. Middle school students experienced higher difference scores from pre to post on these three constructs, when compared to their high school counterparts.

**Table 4:** Level of schooling by gender ANOVA among knowledge difference scores.

Knowledge and understanding domains	Middle school M (SD)	High school M (SD)	*F* value	*p* value	Partial Eta^2^
Establishing & Maintaining Sobriety (*n* = 250)	2.83 (2.67)	1.81 (2.22)	10.99	0.001***	0.043
Conflict Resolution (*n* = 246)	2.77 (2.64)	1.91 (2.08)	7.93	0.005**	0.032
Healthy Relationships (*n* = 225)	2.55 (2.31)	1.84 (2.15)	5.74	0.017**	0.025

***p < 0.001; **p < 0.01.

### Behavior change

As a result of attending the GRL training, nearly 44% of students surveyed reported they would change something within their lives; 45% reported that they would “maybe” change something. The most frequent categories of behavioral change are mental health, followed by behavior, attitude, thinking/perspective, issues with anger, interactions with others, and other (Table 5).

**Table 5:** Qualitative themes related to behavior change resulting from GRL (*n* = 551).

Category	Frequency (*n*)	Exemplar
My Mental Health	34	“I will change what I do if I ever feel suicidal”
My Behavior	19	“I will change my behavior toward close friends and family.”
My Attitude	16	“I will change the attitudes of how people feel about others and transfer to somewhere better place, somewhere where I could focus on my life and future goals.”
My Thinking / Perspective	15	“How I see my perspective on things especially with the things we learned, for example grief, suicidal prevention, and more.”
My Issues with Anger	15	“I will address the anger that remains within me.”
My Interactions with Others	13	“To notice when people are struggling.”

Other categories mentioned include doing better in school, improving health outcomes (eating better, exercising more), involving spirituality and culture, changing “bad habits,” and being a better person/friend.

### GRL legacy impacts

Students were provided a predetermined list of ways that GRL training could impact their lives and told to select all that applied. The most frequent responses are presented in Table 6. Top responses included being more hopeful about the future (45%), feeling more positive (40%), and knowing how to help someone who is suicidal (30%).

**Table 6:** Self-Reported Impacts of GRL Training (*n* = 973).

Impacts	n	%
I am more hopeful about the future	440	45%
I feel more positive	393	40%
I can / know how to help someone who is suicidal	280	30%
I know how to break unhealthy cycles	218	22%
I know more about strategic planning and goal setting	256	26%
I understand the impact of colonization	193	20%
I was not impacted at all	117	12%
I feel more connected to my spirit	153	16%
I know how to address conflicts in a healthy way	152	16%
I know how to forgive and why it is important	160	16%
I understand how to deal with grief	147	15%
I understand the impact of historical trauma and racism on my life	142	15%

## Discussion

GRL trainings significantly improved generational knowledge, holistic wellbeing, and healthy behaviors. This GRL evaluation fills a gap in the existing suicide prevention literature and demonstrates the importance of AI/AN community-level protective factors through culturally centered, community-initiated trainings like GRL. Findings also underscore the importance of early, developmentally tailored interventions (31), holistic community-based training, and upstream prevention approaches that consider collective rather than individual risk (32).

Holistic approaches to wellbeing, conceptualized by the balance of the medicine wheel (or the mental, physical, spiritual, and emotional domains), provide valuable insight to address historical trauma and the lingering impacts of colonization (3). In this study, holistic domains of wellness were all positively correlated with each other; this is consistent with the medicine wheel approach, where one domain is related to another, and the goal is always to find balance within the circle of wellness and return to one’s spiritual center (33). In this study, higher Mental Wellness scores had greater increases in knowledge from pre to post in the areas of Forgiveness & Letting Go, Coping with Trauma, and Establishing & Maintaining Sobriety. Here, coping skills and forgiveness are aspects of mental wellness, and it was expected that as Mental Wellness scores increased, other areas also increased. Sobriety is related to coping skills and the ability to prioritize mental well-being (28). Physical Wellness scores correlated with larger pre to post knowledge gains for Sources of Strength and Conflict Resolution. Higher Spiritual Wellness scores correlated with larger difference scores from pre to post for Colonization, Racism, and the Role of Native People. Additional research is needed to explore the reasons for these differences. Higher Emotional Wellness scores were correlated with larger differences regarding the construct of Healing Shame. Individuals with higher Emotional Wellness scores may be more emotionally ready to heal than those with lower Emotional Wellness scores. Future studies may explore how GRL impacts pre to post knowledge gains but should remain mindful that holistic wellbeing cannot be reduced to words or a numeric value.

The age of participants affected their knowledge scores and the legacy impacts of GRL training. Middle school students reported greater increases in pre- to post-knowledge than high school students. This is consistent with related developmental research (32) that shows middle school-aged students may experience greater impacts from prevention training than high school students (34). High school students showed a greater difference in scores regarding Grief & Loss. Middle school students had larger difference scores for Sobriety & Healthy Relationships. It is possible that middle school students have less knowledge related to establishing sobriety than high school students because, developmentally, they have less experience and exposure to these substances. At the same time, middle school students reported higher mean scores for gains in Healthy Relationship Knowledge. Middle school students may have had fewer relationship issues with their families or significant partners. High school students reported higher mean knowledge scores related to Grief & Loss, which may be attributed to their developmental readiness to receive information on this topic (32) or to the fact that they have lived longer and witnessed the effects of international traumas, violence, and premature death (35).

The legacy impacts of the GRL training over time demonstrate that it positively affects students, increasing their hopefulness about the future and fostering a more positive outlook on their lives. Both are protective factors that may mitigate suicide risk in the future. One-third of the participants know how to help someone who is suicidal because of the GRL training. Students learned about goal setting and felt more connected to their spirits when they left the training. GRL’s spiritual and cultural aspects are not fully described here because they are unique, confidential, and respected within each GRL training community and tribal location. However, GRL’s strong emphasis on culture and spirituality likely strengthens identity and opportunities for community healing (3). Documenting the collective impact of universal prevention approaches like the GRL in AI/AN communities is an area for future work. Tribes could develop programs and strategies that are community-led and evaluated based on a community’s definition of wellbeing and assessed over time, guided by the question, “How many people need to be trained in GRL to strengthen cultural resiliency and improve community conditions for generational healing?”

Behavior change themes demonstrate that GRL elevates protective factors related to suicide, including positive mental health, personal behaviors, attitudes, thinking and perspective, issues with anger, and interactions with others. This is consistent with other research where suicide prevention training reduced hopelessness and increased confidence (21). Nearly half of the American Indian students plan to make behavior changes as a result of attending the GRL training. Future studies could follow up with GRL participants to determine actual behavior changes that resulted from the training and additional support needed.

### Limitations

The results of this study show the positive impacts and significant effects of GRL, but these must be interpreted with caution. First, results may not be generalizable to other AI/AN populations. Participants were recruited using convenience sampling methods, and the results only reflect the responses of participants who could attend the training. Second, the retrospective evaluation design is based on self-reported knowledge gains and how participants feel their knowledge increased as a result of the training. While this method is recommended and accepted for use in this population (36), it is subject to limitations. Additionally, social desirability bias in self-report data may artificially inflate the effect sizes and outcomes of the GRL training, particularly when students want to be perceived as more socially desirable (37). Third, participants were from diverse tribal and community backgrounds and may define suicide and the prevention of suicide in different ways (38). Therefore, the actual uses of GRL skills to strengthen protective factors and prevent suicide in communities may vary; some may rely on cultural connections and knowledge, while others may use personal relationships and connection to elders. Finally, the multi-level impacts of GRL have been conceptualized in this evaluation (see Figure 1) but have not been tested. Additional evaluation and follow-up with GRL students, communities, families, and tribes are necessary to fully understand how GRL impacts communities and how the skills and knowledge acquired are applied outside the GRL training environment.

### Implications

The benefits of generational knowledge and understanding gained from GRL training are far-reaching and go beyond the context of this study. Knowledge and skills gained during the GRL will be shared, passed down, and modeled for future generations. Continued education about culture and collective approaches to health and wellbeing are necessary at all levels, from state and county prevention efforts to federal and national approaches to prevent suicide. Importantly, healing looks different in Tribal communities; the information covered in the GRL facilitates healing. For example, clinicians are on-site to provide additional support and referrals when needed. Cedar or sage is offered. These are culturally centered coping mechanisms that are not typically available in Western psychological treatments and standards. The Good Road of Life conceptual model provides a visual representation of this study’s findings and impacts. Practice recommendations include offering the GRL and other culturally-centered interventions before suicides occur, so that individuals have the skills, resilience, and protective factors available to them when needed. Future research could be conducted to explore how GRL fully impacts individuals and communities weeks, months, and even years after the GRL training. This research could explore the different ways that GRL impacts collective healing (see Figure 1), cultural resilience and spirituality, kinship and relationships, and overall wellbeing.

## Conclusion

“Walking the Good Road of Life” is something that every person on this earth can do if they are given the opportunity. Tribal teachings and traditional roles were severely impacted by colonization. The GRL curriculum elevates culture and kinship systems, connecting students to elders, clans, and kinship systems that traditionally taught them how to live and navigate the world (1). Collective healing is possible, and the authors demonstrate that GRL is effective in addressing the suicide crisis in AI/AN communities.

Individuals who leave this Great Turtle Island by suicide are not forgotten, but we have missed opportunities to give them skills, knowledge, and connections to deal with the conditions they face. One tribe conceptualized suicide as a spirit that comes to warn and help communities (17). The warning signs are everywhere: community conditions, oppression, marginalization, persistent poverty, and generational traumas must be addressed. Legacy impacts and generational knowledge cultivated from the Good Road of Life in Tribal communities are the way forward.

*“Today, talking about suicide made me realize in my darkest moments, I did not wanna die. It was the depression speaking.”—*GRL Student, 2022.

## Data Availability

The raw data supporting the conclusions of this article will be made available by the authors, without undue reservation.

## Footnotes

^1^ www.nativeprideus.org

## Author’s note

This paper and work are dedicated to those we have lost and is for those coming to us, the seventh generation. Our prayer is that GRL will reach all AI/AN people and communities, to walk in beauty on the Good Road of Life.

## Terminology

American Indian/Alaska Native (AI/AN) refers to someone who has blood degree from and is recognized by a federally recognized tribe or village (as an enrolled tribal member) and/or the United States.

American Indian is used when no Alaska Native participants were involved or present in a GRL training.

Native Americans refer to the indigenous peoples of North America who were the original inhabitants of the United States before colonization.

In this study, we use both terms depending on the literature cited and the context of the GRL training.

## References

[1] Sooktis R Littlebird V Parker J Medicine Bull L Tall Bull L Hollow Breast D. Cheyenne cultural kinship systems. Boys and girls Club, northern Cheyenne nation. Lame deer, MT: (2001). Unpublished report for. 1.

[2] Parker T Kelley A. American Indian and Alaska native life expectancy: writing a new narrative. JAMA. (2023) 330:2053–4. doi: 10.1001/jama.2023.22614, PMID: 37930691

[3] Duran E Duran B Heart MYHB Horse-Davis SY. Healing the American Indian soul wound In: International handbook of multigenerational legacies of trauma. Boston, MA: Springer US (1998). 341–54.

[4] Ivey-Stephenson A Trinh E Zhou H Welder L. Circumstances associated with suicides among American Indian/ Alaska native persons–NVDRS, 2015–2020. Inj Prev. (2022) 28:A18–8. 10.15585/mmwr.mm7137a1 PMC9484806 36107803

[5] Curtin SC. Deaths: leading causes for 2021. Natl Vital Stat Rep. (2024) 73. doi: 10.15585/mmwr.mm7137a1 38085308

[6] Price JH Khubchandani J. Suicidal thoughts and behaviors in American Indian and Alaska native adolescents. J Community Health. (2024) 50, 227–234. doi: 10.1007/s10900-024-01411-z, PMID: 39404991 PMC11937164

[7] Schuler A Wedel A Kelsey SW Wang X Quiballo K Beatrice Floresca Y. Suicidality by sexual identity and correlates among American Indian and Alaska native high school students. J Adolesc Health. (2023) 73:1030–7. doi: 10.1016/j.jadohealth.2023.07.020 37737757 PMC10840863

[8] Rey LF Wiglesworth A Prairie Chicken ML Fetter AK Azarani M Riegelman A. A systematic review of research methodologies in American Indian and Alaska native suicide research from 2010 to 2020. Cult Divers Ethn Minor Psychol. (2023) 29:358–71. doi: 10.1037/cdp0000531, PMID: 35225637

[9] Substance Abuse and Mental Health Services Administration. Suicide clusters within American Indian and Alaska native communities: a review of the literature and recommendations. Rockville, MD: Center for Mental Health Services, Substance Abuse and Mental Health Services Administration (2017).

[10] Hirsh J Webb J Jaglic E. Forgiveness, depression, and suicidal behavior among a diverse sample of college students. J Clin Psychol. (2011) 67:896–906. 21633957 10.1002/jclp.20812

[11] Kelley A Small C. Healers need healing too: results from the good road of life training. Am Indian Alaska Native Ment Health Res. (2020) 27:60–75. doi: 10.5820/aian.2702.2020.60, PMID: 33253409

[12] Alcántara C Gone JP. Reviewing suicide in native American communities: situating risk and protective factors within a transactional–ecological framework. Death Stud. (2007) 31:457–77. doi: 10.1080/07481180701244587, PMID: 17554839

[13] Brockie T Kahn-John M Mata Lopez L Bell E Brockie T Brockie T. A mixed-methods study protocol on factors contributing to suicide clusters among native American youth in a northern plains reservation. Front Public Health. (2024) 11, 1–12. doi: 10.3389/fpubh.2023.1281109 PMC10800579 38259800

[14] Qiao N Bell TM. Indigenous adolescents’ suicidal behaviors and risk factors: evidence from the National Youth Risk Behavior Survey. J Immigr Minor Health. (2017) 19:590–7. doi: 10.1007/s10903-016-0443-x, PMID: 27271955

[15] Nock MK Borges G Bromet EJ Cha CB Kessler RC Lee S. Suicide and suicidal behavior. Epidemiol Rev. (2008) 30:133–54. doi: 10.1093/epirev/mxn002, PMID: 18653727 PMC2576496

[16] Suicide Prevention Resource Center (SPRC). (2025). American Indian Alaska native populations. Available online at: https://sprc.org/about-suicide/scope-of-the-problem/racial-and-ethnic-disparities/american-indian-and-alaska-native-populations/ (Accessed on 4-1-25)

[17] Kelley A Small C Small MC Montileaux H White S. Defining cultural resilience to strengthen native youth: a brief report from the intergenerational connection project. Pract Anthropol. (2018) 40:5–9. doi: 10.17730/0888-4552.40.4.5

[18] United States Health and Human Services (USHHS). (2024). National strategy for suicide prevention. Available online at: https://www.hhs.gov/programs/prevention-and-wellness/mental-health-substance-abuse/national-strategy-suicide-prevention/index.html (Accessed on 4-1-25)

[19] Allen J Wexler L Rasmus S. Protective factors as a unifying framework for strength-based intervention and culturally responsive American Indian and Alaska native suicide prevention. Prev Sci. (2022) 23:59–72. doi: 10.1007/s11121-021-01265-0, PMID: 34169406

[20] Wexler L White J Trainor B. Why an alternative to suicide prevention gatekeeper training is needed for rural indigenous communities: presenting an empowering community storytelling approach. Crit Public Health. (2015) 25:205–17. doi: 10.1080/09581596.2014.904039, PMID: 36779086 PMC9909836

[21] LaFromboise TD Malik SS. A culturally informed approach to American Indian/Alaska native youth suicide prevention In: Zane N Bernal G Leong FTL, editors. Evidence-based psychological practice with ethnic minorities: Culturally informed research and clinical strategies. Washington, DC: American Psychological Association (2016). 223–45.

[22] Caughlan C Kakuska A Manthei J Galvin L Martinez A Kelley A. Mind4Health: decolonizing gatekeeper trainings using a culturally relevant text message intervention. Front Public Health. (2024) 12:1397640. doi: 10.3389/fpubh.2024.1397640, PMID: 39286750 PMC11403716

[23] Kingi-Uluave D Taufa N Tuesday R Cargo T Stasiak K Merry S. A review of systematic reviews: gatekeeper training for suicide prevention with a focus on effectiveness and findings. Arch Suicide Res. (2024) 29, 329–346. doi: 10.1080/13811118.2024.2358411, PMID: 38884349

[24] Mueller-Williams AC Hopson J Momper SL. Evaluating the effectiveness of suicide prevention gatekeeper trainings as part of an American Indian/Alaska native youth suicide prevention program. Community Ment Health J. (2023) 59:1631–8. doi: 10.1007/s10597-023-01154-6, PMID: 37558869 PMC10598093

[25] Wexler LM Gone JP. Culturally responsive suicide prevention in indigenous communities: unexamined assumptions and new possibilities. Am J Public Health. (2012) 102:800–6. doi: 10.2105/AJPH.2011.300432, PMID: 22420786 PMC3483901

[26] Kelley A Small C Charani Small M. Responsible fatherhood program for native men: a mixed-method evaluation of the good road of life training. J Fam Strengths. (2020) 20:9. doi: 10.58464/2168-670X.1426

[27] Kowalski MJ. Measuring changes with traditional and retrospective pre-posttest self-report surveys for a brief intervention program. Eval Program Plann. (2023) 99:102323. doi: 10.1016/j.evalprogplan.2023.102323, PMID: 37276793

[28] Kelley A BigFoot DS. Spiritual healing for trauma and addiction: Discussions of mental health, recovery, and faith. Routledge IAbingdon, UK, (2023).

[29] Kuhlman ST Walch SE Bauer KN Glenn AD. Intention to enact and enactment of gatekeeper behaviors for suicide prevention: an application of the theory of planned behavior. Prev Sci. (2017) 18:704–15. doi: 10.1007/s11121-017-0786-0, PMID: 28444519

[30] Clarke V Braun V. Thematic analysis. J Posit Psychol. (2017) 12:297–8. doi: 10.1080/17439760.2016.1262613

[31] Oppenheimer CW Glenn CR Miller AB. Future directions in suicide and self-injury revisited: integrating a developmental psychopathology perspective. J Clin Child Adolesc Psychol. (2022) 51:242–60. doi: 10.1080/15374416 35380885 PMC9840868

[32] Wyman PA. Developmental approach to prevent adolescent suicides: research pathways to effective upstream preventive interventions. Am J Prev Med. (2014) 47:S251–6. doi: 10.1016/j.amepre.2014.05.039, PMID: 25145747 PMC4143775

[33] Kelley A Steinberg R McCoy TP Pack R Pepion L. Exploring recovery: findings from a six-year evaluation of an American Indian peer recovery support program. Drug Alcohol Depend. (2021) 221:108559. doi: 10.1016/j.drugalcdep.2021.108559, PMID: 33548899

[34] Beamon ER Henson RA Kelly SE Hansen WB Wyrick DL. Fidelity of DARE officers’ delivery of “Keepin’it REAL” in elementary & middle school. Prev Sci. (2023) 24:985–98. doi: 10.1007/s11121-023-01548-8, PMID: 37358751 PMC10409848

[35] Willmon-Haque S BigFoot SD. Violence and the effects of trauma on American Indian and Alaska native populations. J Emot Abus. (2008) 8:51–66. doi: 10.1080/10926790801982410

[36] Kelley A BigFoot D Small C Mexicancheyenne T Gondara R. Recommendations from an American Indian reservation community-based suicide prevention program. Int J Hum Rights Healthc. (2015) 8:3–13. doi: 10.1108/IJHRH-10-2013-0025

[37] Krumpal I. Determinants of social desirability bias in sensitive surveys: a literature review. Qual Quant. (2013) 47:2025–47. doi: 10.1007/s11135-011-9640-9

[38] Kelley A Witzel M Fatupaito B. Preventing substance use in American Indian youth: the case for social support and community connections. Subst Use Misuse. (2019) 54:787–95. doi: 10.1080/10826084.2018.1536724, PMID: 30574816

[39] Barzilay S Apter A. (2014). Psychological models of suicide. Archives of suicide research, 18, 295–312. 24568371 10.1080/13811118.2013.824825

[40] Kocherlakota N. (2015). Persistent Poverty on Indian Reservations: New Perspectives and Responses. Federal Reserve Bank of Minneapolis. Available at: https://www.minneapolisfed.org/article/2015/persistent-poverty-on-indian-reservations-new-perspectives-and-responses

[41] National Indian Child Welfare Association (2018). Setting the Record Straight: The Indian Child Welfare Act Fact Sheet. Available at: https://www.nicwa.org/wp-content/uploads/2025/02/Setting-the-Record-Straight-2018.pdf (accessed 6-1-2025).

[42] Blume AK Tehee M Galliher RV. (2019). Experiences of discrimination and prejudice among American Indian youth: Links to psychosocial functioning. Handbook of Children and Prejudice: Integrating Research, Practice, and Policy, 389–404.

