# Supplementary material for: Walking the good road of life: a longitudinal evaluation of American Indian youth suicide prevention training
Source: Front Public Health. 2025 Jun 30;13:1616464. doi: 10.3389/fpubh.2025.1616464 (PMC12258292; doi:10.3389/fpubh.2025.1616464)
Supplement: Supplementary file 1 [file Supplementary_file_1.docx]

**Supplemental File 1**

**Example of GRL Training of Trainers and Agenda**

TRAINING OF TRAINERS-DAY ONE

9:00-9:15 am-------------Registration/Photos Taken/Coffee/Socializing

9:15-9:30 am------------Welcome/Blessing/Introductions/Overview

9:30-9:45 am------------Norms/Expectations

9:45-10:30 am-----------Review GRL curriculum and trainer manual

10:30-11:15 am---------Team Formation

11:15-11:30 am---------BREAK

11:30 - Noon ----------Review roles-responsibilities/trauma informed/Facilitation

12:00-1:00 pm---------Lunch

1:00-1:30 pm----------Team Building

1:30-2:00 pm----------Resources and support for participants-referrals

2:00-2:30 pm---------Strengthening cultural resiliency and emotional intelligence

2:30-2:45 pm---------BREAK

2:45-3-3:30 pm------Open Mic/Evaluation /Debriefing process-Closing

AGENDA-DAY TWO

9:00-9:15 am -------------Registration/Photos Taken/Coffee/Socializing

9:15-9:30 am------------- Welcome/Blessing/Introductions/Overview

9:30-9:45 am------------ Norms/Expectations/Team-Trust Building Skills

9:45-11:00 am---------- Colonization/Racism/and Impact on Native Families

11:00-11:15 am-------- BREAK

11:15—Noon-----------Spirituality and Sources of Strength

12:00 -12:45 pm --------- LUNCH

12:45- 2:00 pm----------New Beginning: Multi-generational Trauma and Breaking Unhealthy

Cycles

2:00-2:15 pm----------- Team-Trust Building Skills

2:15-3:00 pm----------- New Beginning: Multi-generational Trauma and Breaking Unhealthy

Cycles- Continued

3:00-3:30 pm----------- Open Mic/Pluses and Wishes/Evaluation/Announcements/

3:30-4:00 pm-----------Adults Debriefing

AGENDA-DAY THREE

9:00-9:30 am------------ Registration/Coffee/Welcome/Blessing

9:30-11:00 am-----------Hostility and Anger Management

11:00-11:15 am---------BREAK

11:15--Noon-------------Healthy Relationships

12:00-12:45 pm--------LUNCH

12:45-1:00 pm---------Team-Trust Building Skills

1:00-2:30 pm---------Greif and Loss and Suicide Prevention

2:30-2:45 pm---------BREAK

2:45-3:00 pm---------Team-Trust Building Skills-Continued

3:00-3:30- pm---------Open Mic/Pluses and Wishes/Evaluations/Announcements

3:30-4:00 pm--------- Adults Debriefing

AGENDA-DAY FOUR

9:00-9:30 am---------- Registration/Coffee/Welcome/Blessing

9:30-10:30 am---------Forgiveness and Letting Go

10:30-10:45 am--------BREAK

10:45-11:00 am--------Team Building Skills

11:00-Noon-------------Sobriety

12:00-12:45 pm--------LUNCH

12:45-1:00 pm---------Team-Trust Building Skills

1:00-2:00 pm----------Emotional Intelligence

2:00-2:15 pm----------Break

2:15-3:00 pm---------Communication Skills and Making Amends

3:00-3:30 pm---------- Open Mic/Evaluations/Announcements/ Closing

3:30-4:00 pm----------Adults Debriefing

**Example of GRL Data Collection Plan and Daily Evaluations**

**Background**

[Native PRIDE](https://www.nativeprideus.org/) is partnering with Little Wound School and [Allyson Kelley and Associates PLLC](https://www.allysonkelleypllc.com/) to implement GRL training for 20 adults and 120 students at a High School on the Reservation.

**Training/Intervention Dates**

TBD

**Training/Intervention and Goals**

This training aims to strengthen cultural resilience, increase skills and knowledge of strong mental health, and create a sense of hope and community among attendees. The training also addresses the challenge that many young people in the community are children of trauma.

**Data Collection Plan/Purpose**

Implement an evaluation for the September 2024 training. Document what participants learned, their sources of strength, and the knowledge gained related to the topics presented.

**Data Collection Method**

GRL online surveys, video interviews with participants, photos, short interviews with participants on-site, and other administrative documents as required. A survey link and QR code are available for student evaluations (see next page). A student paper survey may be printed for students who cannot use QR codes or online data collection methods.

**Products**

GRL training evaluation report (5–10-page summary). Native PRIDE, AKA, and other partners will share results via a landing page, website, funding agency reports, and other recommended methods.

**Data Collection Directions and Explanations**

Administer GRL evaluation at the end of the day using QR codes provided

**Evaluation Team**

For more information about this plan, contact [ak@allysonkelleypllc.com](mailto:ak@allysonkelleypllc.com)

**Example of Evaluation QR Codes and Bitlys**

**Student Day 1**

<https://corexmsvpyqcnf4wblhy.sjc1.qualtrics.com/jfe/form/SV_20uXTLFGmkjHaPs>

Bitly: <https://bit.ly/WYIHS_Day1>


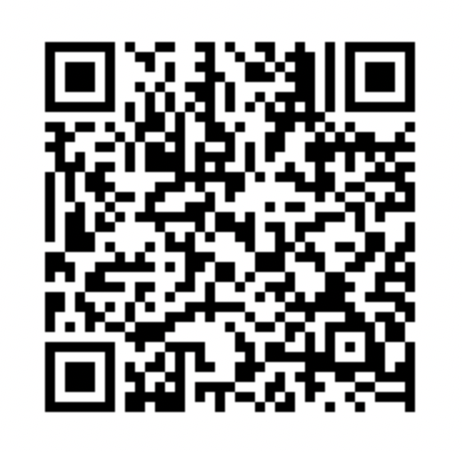


**Student Day 2**

<https://corexmsvpyqcnf4wblhy.sjc1.qualtrics.com/jfe/form/SV_6icsXiC2rbObNr0>

Bitly: <https://bit.ly/WYIHS_Day2>


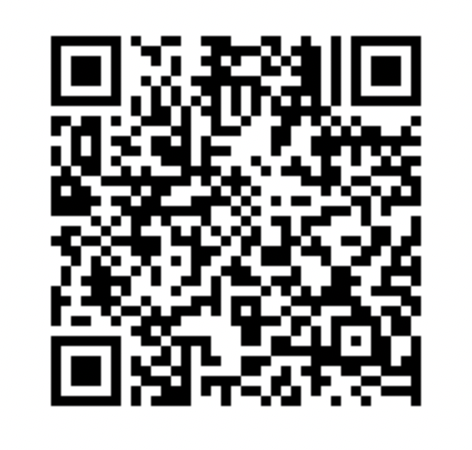


**Student Day 3:**

<https://corexmsvpyqcnf4wblhy.sjc1.qualtrics.com/jfe/form/SV_6Ls6CSU3YYKtt8q>

Bitly: <https://bit.ly/WYIHS_Day3>


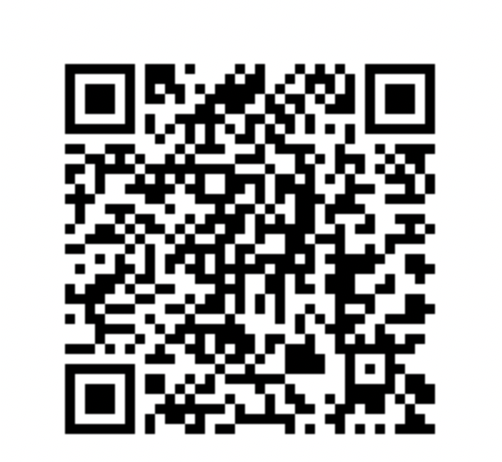


**Training of Trainers:**

<https://corexmsvpyqcnf4wblhy.sjc1.qualtrics.com/jfe/form/SV_da0zMY9IkuCeeW2>

Bitly: <https://bit.ly/WYIHS_TOT>


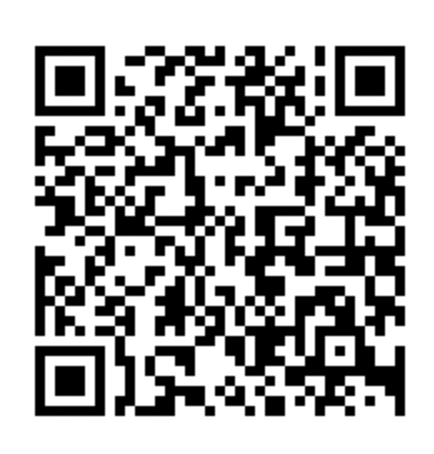


**Example Printed Daily GRL Evaluations**

Gender:____________ Age:______________

1. Was today’s GRL program easy to follow and understand?

Yes Not Sure No

1. The facilitators were organized, knowledgeable, and supportive?

Strongly Disagree Disagree Neither Agree Strongly Agree

1. The session activities were meaningful and relevant to me.

Strongly Disagree Disagree Neither Agree Strongly Agree

1. I will apply what I learned from today’s training in my life

Yes Not Sure No

1. How will you apply what you learned?

_____________________________________________________________________

1. Select the statements that best describe you. Using a scale of 1 to 10 please rate your understanding BEFORE attending the GRL and AFTER. 1 is No Understanding and 10 is Complete Understanding.

|  | Knowledge Before | Knowledge After |
| --- | --- | --- |
| Colonization, Racism, and the Impact on Native People | 1 2 3 4 5 6 7 8 9 10 | 1 2 3 4 5 6 7 8 9 10 |
| Multigenerational Trauma and Breaking Unhealthy Cycles | 1 2 3 4 5 6 7 8 9 10 | 1 2 3 4 5 6 7 8 9 10 |
| Grief-Loss | 1 2 3 4 5 6 7 8 9 10 | 1 2 3 4 5 6 7 8 9 10 |
| Suicide Prevention | 1 2 3 4 5 6 7 8 9 10 | 1 2 3 4 5 6 7 8 9 10 |

1. How did the GRL training impact you? Select ALL that apply

- I understand the impact of colonization
- I feel more connected to my spirit
- I understand the impact of historical trauma and racism on my life
- I know how to break unhealthy cycles
- I understand how to deal with grief
- I know the suicide risk factors and how to get help
- I can help someone who is suicidal
- I am more knowledgeable about being trauma informed
- I was not impacted at all
- Other:________________________________________________________________

1. What do you still want to know?

_______________________________________________________________________

**Supplemental Table 5. Qualitative Themes Related to Behavior Change Resulting from GRL (N = 551)**

What will you change?

Top Category- Mental Health (34)

- - "If I ever feel suicidal, I will get help"
  - “Accepting feedback better, handling my emotions better and always smudging and praying.”
  - “Avoid jumping into conclusions and to not judge other people by their appearance, the way they think and talk. Because they might be into something.”
  - “Coping strategies”
  - “Emotions and move to a good path”
  - “Handling my emotions”
  - “Helping better with people struggling with mental health and drug abuse.”
  - “How I manage to get through things with my emotions”
  - “How to find signals of someone depressed”
  - “How to break unhealthy habits”
  - “Probably my grief, or worries.”
  - “Start helping those who are impacted by suicide”
  - “I will change what I do if I ever feel suicidal”
  - “Manage how I am feeling and what I can do to control them.”
  - “I will be more understanding and empathetic to other people because i know that everyone has their own problem or struggles.”

My behavior (19)

- - “My behavior and setting goal”
  - “My behavior”
  - “My behavior”
  - “My behaviour”
  - “My behaviour towards close friends and family”
  - “Probably start being humble and nice trustful and understanding and kind even rooting”
  - “I will change my behavior”
  - “I will change my ways”
  - "Doing my chores more"
  - “My way of being”
  - “BEHAVIOR”
  - “Behavior and my anger problems.”
  - “Behavior and my chewing habits of plastic😝”
  - “Behavior wise”
  - “Behavior”
  - "My behavior"
  - “Be less quiet”

My attitude (16)

- - “My attitude and be nice to everyone and lastly show respectful language”
  - “I will change the attitudes of how people feel about other and transfer to somewhere better place, somewhere I could focus on my life and future goals.”

My thinking / perspective (15)

- - “My perspective towards life.”
  - “What I would change from the training and skills I learned today to my everyday life would be how I see my perspective on things especially with the things we learned, for example grief, suicidal prevention, and more.”
  - “I will change how I see and think about other people when I don’t know them or their story.”
  - “I will change on how i see myself and how I think of others”
  - “I will change on how to be a better person to myself and others around me who I don’t know what’s going on in their life”
  - “I will be more positive and careful around others about my actions and words”

Issues with Anger (15)

- - “Control my anger and be patient with myself and other people.”
  - “My hostility”
  - “Not to be mean to my friends”
  - “The way I deal with my anger and my hostility”
  - “I will address the anger that remains within me.”
  - “I will be nicer to people instead of being mean to my friends”
  - “What I will change is my understanding of people and being more positive”

Interactions with Others (13)

- - “Change the way I talk and to respect others
  - “The way I act towards people and how I handle situations
  - “My actions towards everyone, regardless the person.
  - “To notice when people are struggling
  - “My relationships and how to talk and communicate in the relationships.
- Other categories included:
  - Doing better in school, improving health outcomes (eating better, exercising more), involving spirituality and culture, changing “bad habits” (cursing), being a better person/friend.

(Positive) Qualitative Feedback

- “I feel more connected to my spirit.”
- “I had a great experience today and nothing could’ve been better.”
- “I just loved it so nothing really”
- “I learned a lot today”
- “I learned more and how to deal with things”
- “I enjoyed all parts of it. Keep it up.”
- “I feel a lot more relaxed”
- “I like how we all can work together and have fun also that we get to learn a little more every day we get to see him”
- “I like the activities because they help me learn more about my culture.”
- “I like the stuff we did at school and all the other stuff that will happen”
- “I liked everything we did”
- “I liked the activities; they are fun and relatable to our culture.”
- “I liked the sweet dance, but I wanted more powwow music to play”
- “I really liked today it was entertaining and fun”
- “I think it is pretty cool. And it is fun to do.”
- “I think today was really good and that it was really fun so I do not have any suggestions today.”
- “It helped a lot I guess I threw away bad memories that I couldn't hold anymore”
- “It was a great experience”
- “It was a really good day and has a lot of impacted on me”
- “It was already good I had fun with this camp I really looking forward to coming next year”
- “It was awesome, super fun yesterday and today”
- “It was good, had a lot of fun”
- “It was great!”
- “It was really fun and enjoyable”
- “It was really good, had fun.”
- “It was very cool. no suggestions”
- “It was wonderful”
- “It would be a good time to explain the way of life”
- “I thought it was quite encouraging and helpful and very spiritual”
- “It helped me and it was emotional for me”
- “It was a great day”
- “It was fun and helped me learn a lot.”
- “It was perfect and fun and I wanna have another thing like this again!”
- “They explained well and they were really supportive and caring”
- “This experience is perfect!! ^_^”
- “This training is perfect ^_^”
- “Today helped me learn a lot”
- “Today I learned a lot about mental health wellness”
- “No today was a good day”
- “Everything is great”
- “It was all awesome”
- “Today talking about suicide made me realize in my darkest moments I didn't wanna die. It was the depression speaking. So, no I do not have any suggestions.”
- “Today was good, but sad. I had to talk about my mom's death.”
